# Synthesis, Characterization and Biological Studies of Metal(II) Complexes of (3*E*)-3-[(2-{(*E*)-[1-(2,4-Dihydroxyphenyl)ethylidene]amino}ethyl)imino]-1-phenylbutan-1-one Schiff Base

**DOI:** 10.3390/molecules20069788

**Published:** 2015-05-27

**Authors:** Ikechukwu P. Ejidike, Peter A. Ajibade

**Affiliations:** Department of Chemistry, Faculty of Science and Agriculture, University of Fort Hare, P.B. X1314, Alice 5700, South Africa

**Keywords:** 2′,4′-dihydroxyacetophenone, unsymmetrical tetradentate Schiff base, antibacterial activity, DPPH, ABTS

## Abstract

Co(II), Ni(II), Zn(II) and Cu(II) complexes of (3*E*)-3-[(2-{(*E*)-[1-(2,4-dihydroxyphenyl)ethylidene]amino}ethyl)imino]-1-phenylbutan-1-one (DEPH_2_) derived from ethylenediamine, 2′,4′-dihydroxyacetophenone and 1-phenylbutane-1,3-dione have been synthesized and characterized by elemental analysis, FTIR, UV-Visible spectroscopy, and screened to establish their potential as antibacterial agents, antioxidants and DPPH radical scavengers. The FTIR spectra showed that the ligand behaves as a dibasic tetradentate ligand with the dioxygen-dinitrogen donor atom system oriented towards the central metal ion. The analytical and spectroscopic data suggest a square planar geometry for Cu(II) and Ni(II) complexes and an octahedral geometry for the Co(II) complex. The ligand and their metal complexes were screened for antibacterial activity against Gram (+) and Gram (−) bacteria by the agar well diffusion method. In addition, the antioxidant activities of the complexes were also investigated through their scavenging effect on DPPH and ABTS radicals. The obtained IC_50_ value of the DPPH activity for the copper complex (2.08 ± 0.47 µM) and that of the ABTS activity for the copper complex (IC_50_ = 2.11 + 1.69 µM) were higher than the values obtained for the other compounds.

## 1. Introduction

Metal complexes of Schiff base derived from the reaction of substituted salicylaldehydes with aliphatic and aromatic amines represent a series of compounds containing nitrogen, sulphur and oxygen ligand donor atoms that has been widely studied [1,2]. Schiff base molecules afford potential sites for bio-chemically active compounds that are related to intermolecular hydrogen bonding and proton transfer equilibria [3]. Metal complexes of Schiff base ligands possess a variety of applications in the biological, analytical, clinical, and industrial areas [4]. In recent times, transition metal complexes of Schiff base ligands have gained considerable attention, not only due to their spectroscopic properties and applications [5] but also due to their remarkable antifungal, antibacterial and antitumor activities [6]. With respect to the biological activity and desirable physicochemical, stereochemical, electrochemical, structural and catalytic properties of Schiff base metal complexes, their values has attracted significant attention and is also relevant for their application as tools for the analysis of pharmacological constituents. Tetradentate Schiff base complexes have been established to form stable complexes, with coordination taking place through the dinitrogen-dioxygen donor atoms [7]. Biological reactions which are essential to life processes usually involve transition metals; these metals usually coordinate with O- or *N*-terminals from proteins in a variety of modes and play a vital crucial role in the conformation and function of biological macromolecules [8]. 1-Phenyl-1,3-butanedione is a flavouring and fragrance agent with a balsamic odour used in foods requiring a balsamic, amber, heather, oriental, or vanilla taste. Nickel(II) and copper(II) complexes of 2-substituted-1-phenyl-1,3-butanedione (R = Cl, NO_2_) and their 2,2′-bipyridine and 1,10-phenanthroline adducts have been reported [9]. Metal coordination complexes have been widely studied for their antimicrobial and anticancer properties [10].

Free radicals have been implicated in the causation of several oxidative damages diseases such as liver chirrhosis, atherosclerosis, cancer, diabetes, ageing. An antioxidant can be defined as any substance that when present at low concentrations compared with those of an oxidizable substrate [11] can inhibit the oxidation of lipids or other molecules by preventing the initiation or propagation of oxidative chain reactions and can thus prevent or repair the damage done to the body’s cells by oxygen [12]. Metal-based antioxidants have gained attention recently for their capacity to protect organisms and cells from damage induced by oxidative stress or scavenge free radicals [13]. These metal complex derivatives which show considerable biological activity, may represent an interesting approach for designing new antibacterial drugs. It is worthy to note there have been extensive studies on the preparation of many symmetrical tetradentate bis-type Schiff bases of 1,2-diamines with *o*-hydroxy aldehydes/ketones [14,15], yet little attention has been focused on the preparation of unsymmetrical tetradentate Schiff bases obtained from 1,2-diamines and different aldehydes, and also those derived from aromatic 1,2-diamines have been underinvestigated [15,16]. From a literature search, it appears that no work has been done on Ni(II), Co(II), Co(II) and Zn(II) metal complexes of the asymmetrical Schiff bases derived from aliphatic 1,2-diamines, 2′,4′-dihydroxyacetophenone and 1-phenyl-1,3-butanedione.

In an effort towards the development of metallodrugs as chemotherapeutic agents with interesting biological properties such as antimicrobial activity playing an important role in protecting the body against damages caused by reactive oxygen species, we report herein the synthesis and characterization of metal complexes of (3*E*)-3-[(2-{(*E*)-[1-(2,4-dihydroxyphenyl)ethylidene]amino}ethyl)imino]-1-phenylbutan-1-one derived from ethylene-1,2-diamine, 2′,4′-dihydroxyacetophenone, and 1-phenyl-1,3-butanedione with Cu(II), Zn(II), Co(II) and Ni(II), and the biological activity of the free ligands and metal complexes against some selected bacterial strains and their ability to scavenge DPPH and ABTS free radicals.

## 2. Results and Discussion

### 2.1. General

The syntheses of the Schiff base and its complexes may be represented by the equation below:

M(CH_3_COOH)_2_·*n*H_2_O + DEPH_2_ → M(DEP) + 2CH_3_COOH + *n*H_2_O

M = Cu(II), Zn(II) (*n* = 2); M = Ni(II), Co(II) (*n* = 4)

**Figure 1:**
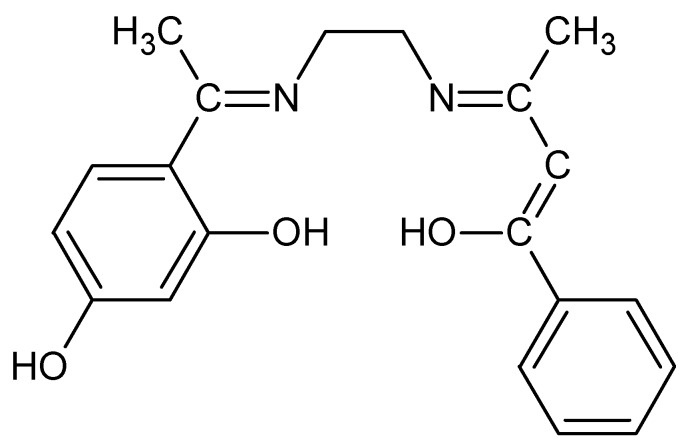
Structure of Schiff base ligand (DEPH_2_).

**Figure 2:**
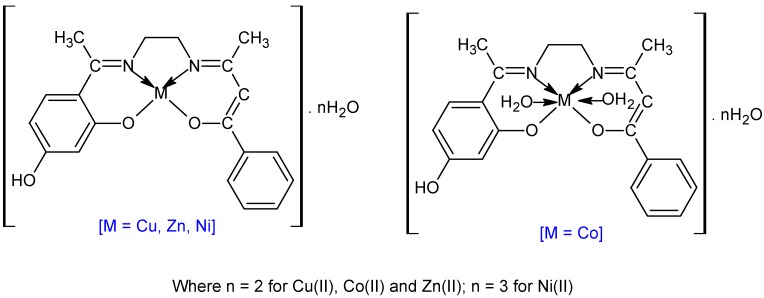
Structure of Schiff base metal complexes.

The structure of the ligand is given in Figure 1. The isolated compounds are coloured powders, stable in air, non-hygroscopic in nature and insoluble in water, non-soluble in other common solvents but easily soluble in DMF and DMSO. The melting points show that all the complexes decomposed before melting. The analytical data (Table 1) indicate that the metal to ligand ratio is 1:1 for all the complex systems. The elemental analysis data of the Schiff base and its metal complexes are in good agreement with the calculated results from the empirical formula for each complex (Figure 2).

**Table 1:** Micro-analytical data and physical properties of the ligand [DEPH_2_] and its metal complexes.

Compounds	Empirical Formula	F. Wt (Grams)	Colour	Yield (%)	% Found (Calcd).			Decomp. Temp, °C	Conductance (µS∙cm^−1^)
					C	H	N		
DEPH_2_	C_20_H_21_N_2_O_3_	337.40	Orange-brown	74.17	70.96(71.20)	5.13(5.27)	8.09(8.30)	211	-
[Zn(DEP)]·2H_2_O	C_20_H_23_N_2_O_5_Zn	436.79	Lemon brown	64.80	55.18(55.00)	4.98(5.31)	6.63(6.41)	189	3.26
[Cu(DEP)]·2H_2_O	C_20_H_23_N_2_O_5_Cu	434.96	Brownish-purple	83.85	55.09(55.23)	5.52(5.33)	6.20(6.44)	209	2.85
[Ni(DEP)]·3H_2_O	C_20_H_25_N_2_O_6_Ni	448.12	Darkish-yellow	73.20	53.81(53.61)	5.48(5.62)	6.33(6.25)	210	4.31
[Co(DEP)(OH_2_)_2_]·2H_2_O	C_20_H_27_N_2_O_7_Co	466.38	Darkish-green	52.90	51.27(51.51)	6.09(5.84)	6.20(6.01)	199	6.70

### 2.2. Infrared Spectra

In order to study the binding mode of the Schiff base (DEPH_2_) to the metal ion in the complexes, the IR spectrum of the free ligand was studied and assigned on the basis of careful comparison of the complex spectra with that of the free ligand. The characteristic IR bands of the ligand and its complexes (KBr pellets, cm^−1^) are given in Table 2. The infrared spectrum of the DEPH_2_ show characteristic bands at 3479 cm^−1^ attributed to the phenolic hydroxyl group, while the characteristic absorptions at 3076 and 2981, 1605, 1543 and 1470, 1288 and 1241 cm^−1^ can be assigned to Ѵ_(CH3/CH2)_, Ѵ_(C=N)_, Ѵ_(C=C)_, Ѵ_(C-O)_, respectively [17,18]. The absence of a strong broad band in the 3479 cm^−1^ region, which is observed in the spectra of the metal complexes, is an indication of deprotonation of the intramolecular hydrogen bonded OH group on complexation and subsequent coordination of the phenolic oxygen to the metal ion [1]. This is further supported by the upward shift to an extent of 48–61 cm^−1^ in the phenolic Ѵ_(C-O)_ [15] band [19]. This shift further confirms the participation of the enolic oxygen in C-O-M bond [20]. The strong band observed at 1605 cm^−1^ in the spectra of the free Schiff base (DEPH_2_) is characteristic of the azomethine Ѵ_(C=N)_ stretching vibration band; this vibration underwent a bathochromic shift to a lower frequency 1584–1601 cm^−1^ upon complexation (Table 2), indicating the bonding of the nitrogen atom of the azomethine group of the free ligand to the metal ions [18,21]. This shift can further be explained by the donation of electrons from nitrogen to the empty d-orbitals of the metal atom [2]. The metal complexes show a broad band in the region 3389–3406 cm^−1^ and a new band at ~860 cm^−1^ that may be assigned to the O-H stretching vibration and out of plane bending of water molecules coordinated to the complexes [17,22,23]. The ring skeletal vibrations (C=C) were consistent in all derivatives and they were not altered upon complexation. In the lower frequency region, some new bands have been observed between 502–536 cm^−1^ that are attributed to Ѵ_(M-N)_ and those within the 427–466 cm^−1^ band are assigned to Ѵ_(M-O)_ [19,20]. The IR spectral information thus supports the suggestion of coordination of the imino nitrogen and phenolic oxygen atoms to the transition metal ions.

**Table 2:** FTIR Spectral data of the Schiff base [DEPH_2_] and its metal complexes.

Compound	Ѵ_(OH)_·Ѵ_(H2O)_	Ѵ_(CH3/CH2)_	Ѵ_(C=N)_	Ѵ_(C=C)_	Ѵ_(C-O)_	Ѵ_(M-N)_	Ѵ_(M-O)_
DEPH_2_	3479mb	3076w, 2981w	1605vs	1543s, 1470s	1288s, 1241s		
[Zn(DEP)]·2H_2_O	3398mb	3074w, 2977s	1601vs	1542s, 1469s	1287s, 1240s	506w	435m
[Cu(DEP)]·2H_2_O	3405mb	3141w, 2976m	1592vs	1515s, 1483s	1240s, 1180m	537m	467m
[Ni(DEP)]·3H_2_O	3389mb	2977s, 2904s	1597vs	1516s, 1485m	1240s, 1180m	559m	458w
[Co(DEP)(OH_2_)_2]·_2H_2_O	3406mb	2975s, 2902m	1598vs	1542m, 1479m	1248s, 1190m	545w	438m

Abbreviations: s = strong; b = broad; v = very; m = medium; w = weak.

### 2.3. Electronic Absorption Spectral and Conductivity Measurements

The electronic spectra of the DEPH_2_ ligand and its metal complexes were recorded in DMF medium at room temperature. The nature of the ligand field around the metal ion has been deduced from the electronic spectra. The bands at 329, 339 and 378 nm are attributable to intraligand π-π* and n-π* transitions (Table 3). In the electronic spectra of the complexes, the intraligand transitions are slightly shifted as a result of coordination in the region at 374–380 nm. The Co(II) complex exhibits three spin allowed transitions at 406, 609 and 682 nm assignable to ^4^T_1g_ (F) → ^4^T1_g_ (P) (ν_3_), ^4^T_1g_ (F) → ^4^A_2g_ (F) (ν_2_) and ^4^T_1g_ (F) → ^4^T_2g_ (F) (ν_1_) transitions respectively, suggesting an octahedral environment around the cobalt ion [2,8]. The electronic spectrum of Ni(II) complex exhibited two absorption bands at 433 and 568 nm which may be assigned to two spin allowed transitions, ^1^A_1g_ → ^1^A_2g_, ^1^A_1g_ → ^1^B_1g_, respectively characteristic of square planar geometry around Ni(II) ion [24]. The spectra of Cu(II) complexes show two bands in the visible region at about 556 and 401 nm, which can attributed to ^2^B_1g_ → ^2^A_1g_ and ^2^B_1g_ → ^2^E_g_ transitions, respectively. The broad band centered at 559 nm is due to the Jahn-Teller distortion, indicating the square-planar geometry around the Cu(II) ion [25]. The Zn(II) complex is diamagnetic as expected and its geometry is most probably similar to the Ni(II) and Cu(II) complexes of the DEPH_2_ ligand. The metal chelate solution in DMF for ~10^−3^ M show low conductance, ranging from 2.85–6.70 µS·cm^−1^ and this supports the non-electrolyte nature of the complexes at room temperature [26].

**Table 3:** Electronic absorption data, assignments of the DEPH_2_ ligand and its metal complexes.

Compounds	Empirical Formula	Electronic Transition, λ_max_ (nm, DMF)	Band Assignments
DEPH_2_	C_20_H_21_N_2_O_3_	329, 339, 378	π-π*, π-π*, n-π*
Zn(DEP)	C_20_H_23_N_2_O_5_Zn	320, 368, 379, 413	π-π*, π-π*, n-π*, L → M (LMCT)
Cu(DEP)	C_20_H_23_N_2_O_5_Cu	322, 362, 380, 401, 558	π-π*, n-π* (LMCT), ^2^B_1g_ → ^2^E_g_, ^2^B_1g_ → ^2^A_1g_
Ni(DEP)	C_20_H_25_N_2_O_6_Ni	321, 354, 374, 433, 568	π-π*, L → M (LMCT), ^1^A_1g_ → ^1^A_2g_, ^1^A_1g_ → ^1^B_1g_
Co(DEP)	C_20_H_27_N_2_O_7_Co	318, 357, 377, 406, 609, 682	^4^T_1g_ (F) → ^4^T1_g_ (P), ^4^T_1g_ (F) → ^4^A_2g_ (F), ^4^T_1g_ (F) → ^4^T_2g_ (F)

### 2.4. Antioxidant Activity

It is well documented that reactive oxygen species (ROS) are involved in the etiopathogenesis of numerous chronic diseases such as atherosclerosis, hypertension and coronary heart disease [27]. These free radicals are produced under certain environmental conditions and during normal cellular functions in the body. Antioxidants thus play an important role to protect the human body against damage by reactive oxygen species. The ability of Schiff bases and their metal complexes to scavenge free radicals is an important property. Different modes of action such as being free radical terminators, chelators of metal ions involved in catalyzing lipid oxidation or oxygen scavengers that react with oxygen closed systems have been used in categorizing antioxidants [28]. In this study, we present the DPPH and ABTS scavenging ability of the Schiff base DEPH_2_ and the corresponding Zn(II), Ni(II), Cu(II) and Co(II) complexes. The antioxidant assay was carried out using different concentrations of the test samples, while ascorbic acid (vitamin C), rutin and butylated hydroxytoluene (BHT) were used as standards.

#### 2.4.1. DPPH Radical Scavenging Activity Assay

The scavenging activity of a chemical/or compound on the DPPH radical as a fast and reliable parameter to measure the *in vitro* antioxidant activity of such sample have been used by diverse researchers [29]. This assay is based on the measurement of the decrease in molar absorptivity of DPPH at 517 nm after reaction with the test compound. The effect of antioxidants on DPPH radical scavenging is due to the hydrogen donating ability or radical scavenging activity of the samples [28].

The scavenging reaction between (DPPH) and an antioxidant (H-D) can be written as:

$$\underset{\text{(Purple)}}{\text{(DPPH)}+\text{(H-D)}}\to \underset{\text{(Yellow)}}{\text{DPPH-H}+\text{(D)}}$$

**Figure 3:**
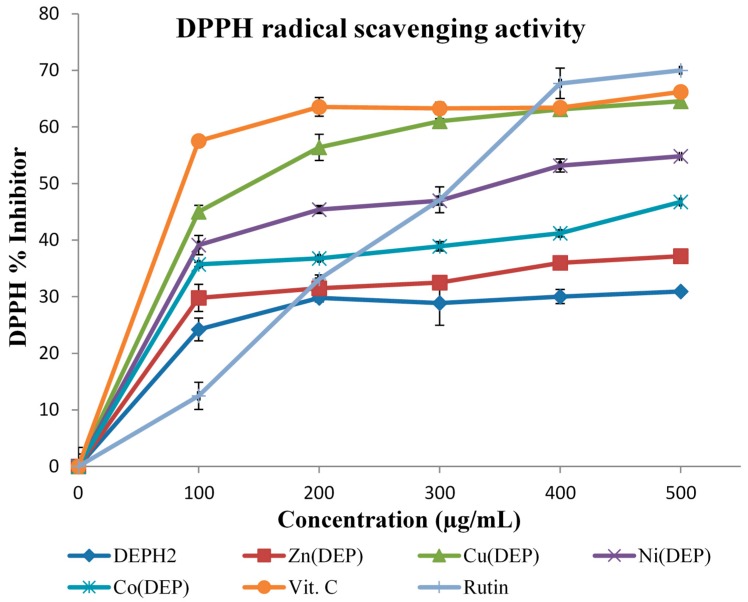
DPPH scavenging activity of DEPH_2_ and DEPH_2_-M(II) complexes.

Antioxidants react with DPPH, a stable free radical that is thus reduced, and as a result the absorbance decreases due to the formation of the DPPH-H from the DPPH radical. The degree of discoloration indicates the scavenging potential of the antioxidant compounds or samples in terms of hydrogen donating ability [28]. Figure 3 shows the dose-response curve of DPPH radical scavenging activity of the Schiff base DEPH_2_ and its Zn(II), Ni(II), Cu(II) and Co(II) complexes, compared with BHT and ascorbic acid. It was observed that metal(II) complexes had higher activity than that of the free Schiff base DEPH_2_. At the lowest concentration (100 µg/mL) the antioxidant activity of the free ligand was found to be 24.20% but, upon complexation, it increased significantly within the range 29.80%–45.01% from Zn(DEP) to Cu(DEP) (Figure 3). 

The increased antioxidant activity of these complexes (Table 4) can be attributed to the electron withdrawing effect of the Zn(II), Ni(II), Cu(II) and Co(II) ions which facilitates the release of hydrogen to reduce the DPPH radical [13]. This proton release were very pronounced in Cu(DEP), with an IC_50_ value of 2.08 ± 0.47 µM, followed by Ni(DEP) [2.52 ± 1.15 µM], Co(DEP) (3.04 ± 0.59 µM) and Zn(DEP) (3.64 ± 1.65 µM). The DPPH radical scavenging ability of the test samples can thus be ranked in the order Vit. C > Cu(DEP) > Ni(DEP) = Rutin > Co(DEP) > Zn(DEP) > Schiff base (DEPH_2_).

**Table 4:** The influence of investigated DEPH_2_, its metal(II) complexes and standard drugs for DPPH * and ABTS * free radicals.

Compounds	DPPH Radical Scavenging Activity		ABTS Radical Scavenging Activity	
	IC_50_ (µM)	*R*^2^	IC_50_ (µM)	*R*^2^
DEPH_2_	4.24 ± 1.23	0.988	1.98 ± 1.55	0.876
Zn(DEP)	3.64 ± 1.65	0.992	2.77 ± 0.74	0.964
Cu(DEP)	2.08 ± 0.47	0.996	2.11 ± 1.60	0.925
Ni(DEP)	2.52 ± 1.15	0.995	3.14 ± 1.78	0.84
Co(DEP)	3.04 ± 0.59	0.985	3.37 ± 0.78	0.975
Vit. C *	1.92 ± 0.63	0.976	-	-
Rutin *	2.52 ± 1.69	0.768	2.83 ± 1.84	0.983
BHT *	-	-	1.64 ± 1.54	0.919

(*n* = 3, X ± SEM), IC_50_- Inhibitory concentration; When the percent inhibition of the tested compounds was 50%, the tested compound concentration was IC_50_. *R*^2^ = correlation coefficient. * Standards.

The enhanced inhibition displayed on the DPPH radical by the test samples shows that the compounds are capable of donating electrons to neutralize free radicals and thus, could be a promising therapeutic agents for the treatment of pathological diseases and conditions caused as a result of excessive radicals or stress.

#### 2.4.2. 2,2′-Azinobis-3-ethylbenzothiazoline-6-sulfonic Acid (ABTS), Radical Scavenger Activity

**Figure 4:**
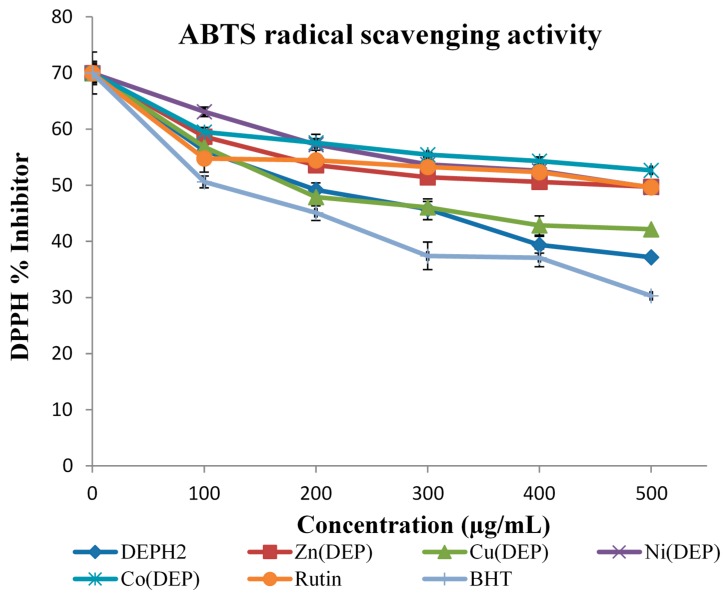
ABTS radical scavenger activity of DEPH_2_ and DEPH_2_-M(II) complexes.

The Schiff base and its complexes were also screened for free radical scavenging activity by the ABTS method [30]. The percentage inhibition results of free radical scavenging activity of the test samples are shown in Figure 4. The coordination of metal ions to the Schiff base DEPH_2_ resulted in a higher spectrum of activity comparable to those of the standards drugs used (rutin and BHT). The test samples exhibited moderate to higher % inhibition scavenging activity than rutin and BHT (standards) at the lowest concentration (100 µg/mL). IC_50_ values of the test samples are listed in Table 4, with Cu(DEP) possessing the highest potency (IC_50_ = 2.11 + 1.69 µM) followed by the zinc, nickel and cobalt complexes. The ABTS radical scavenging ability of the test samples can be ranked in the order: BHT > DEPH_2_ > Cu(DEP) > Zn(DEP) > Rutin > Ni(DEP) > Co(DEP). Furthermore, the synthesized compounds scavenged the DPPH radical in a concentration-dependent manner.

### 2.5. In Vitro Antimicrobial Studies

The results of *in vitro* antibacterial activity of the Schiff base DEPH_2_ and its Co(II), Ni(II), Cu(II) and Zn(II) complexes are presented in Table 5. Amoxicillin and ciprofloxin were used as positive standards and DMSO was used as negative control for these antibacterial activities. In general, metal(II) complexes have been shown to be more effective than the free ligands and the same was observed in this study, *i.e*., that the complexes are more active than the parent Schiff base ligand. The Cu(II) complex was found to show bactericidal activity against *B. cereus*, *S. aureus* and *S. flexineri* and bacteriostatic activity against *S. faecalis*, *P. aeruginosa*, and *E. coli*. A fairly moderate effectiveness is exhibited by Ni(II) complex, which acts within the MIC range of 5–10 mg/mL towards *B. cereus*, *S. aureus*, *S. flexineri* and *E. coli*. The Co(II) and Zn(II) complexes show less activity towards *B. cereus* and *S. aureus*, respectively, but had no activity against the organisms *S. faecalis*, *P. aeruginosa*, *S. flexineri*, *E. coli* within the range of concentrations considered. However, these compounds could be active against the organisms at concentrations greater than 10 mg/mL (MIC > 10 mg/mL). The *in vitro* antibacterial activity results (Table 5) revealed that the ligand was bacteriostatic against all bacterial strains except *S. aureus*.

The activity order of the synthesized compounds is as follows: Cu(II) > Ni(II) > Co(II) > Zn(II) > DEPH_2_. The higher activity of the metal complexes may be due to the effect of metal ions on the normal cell membrane. The variation in the activity of different complexes against different organisms depends either on the impermeability of cells of the microbes or differences in the ribosomes of microbial cells [31].

The higher antibacterial activity of Schiff base metal complexes than the free Schiff base ligands can be explained by chelation of the Schiff base with metal ions [3] as metal chelates display both polar and nonpolar properties; this makes them suitable for permeation into cells and tissues. The polarity of the metal ion will be reduced to a greater extent due to the overlap of the ligand orbital upon chelation, and partial sharing of the positive charge of the metal ion with donor groups. Chelation increases the delocalization of π-electrons over the entire chelate ring and enhances the penetration of the complexes into lipid membranes [2,32]. It also increases the hydrophilic and lipophilic nature of the central metal ions, probably leading to liposolubility and permeability through the lipid layer of cell membranes. Further, lipophilicity, which controls the rate of entry of molecules into the cell, is modified by coordination, so the metal complex can become more active than the free Schiff base ligand [33]. However, when the antimicrobial activity of the Schiff base DEPH_2_ and its metal complexes are compared to the standards, the activity exhibited by the ligand and the metal complexes was lower.

**Table 5:** Minimum inhibitory concentration (MIC) values (mg/mL) of the Schiff Base ligand DEPH_2_ and its metal complexes.

Compounds	Gram Positive Bacteria			Gram Negative Bacteria		
	S. *faecalis*	*B. cereus*	*S. aureus*	*P. aeruginosa*	*S. flexineri*	*E. coli*
DEPH_2_	<10	<10	10	<10	<10	<10
Co(DEP)	<10	10	<10	<10	<10	<10
Ni(DEP)	<10	10	5	<10	10	10
Cu(DEP)	10	5	2.5	<10	5	10
Zn(DEP)	<10	<10	10	<10	<10	<10
Amoxicillin ^a^	1.25	0.312	1.25	1.25	1.25	0.625
Ciprofloxacin ^a^	0.312	0.312	0.312	0.312	0.312	0.312

^a^ Standards.

## 3. Experimental Section

### 3.1. Materials

All reagents used were of analytical grade and are used as received. Cu(II)/Zn(II)/Co(II)/Ni(II) acetate, ethylenediamine and ascorbic acid were from Merck (Johannesburg, South Africa). 2′,4′-Dihydroxy-acetophenone and 1-phenylbutane-1,3-dione were from Aldrich (Johannesburg, South Africa) and used as received. 1,1-Diphenyl-2-picrylhydrazyl (DPPH), 2,2′-azinobis-3-ethylbenzothiazoline-6-sulfonic acid (ABTS), rutin hydrate, and butylated hydroxytoluene (BHT), were purchased from Sigma Chemical Co. (St. Louis, MO, USA).

### 3.2. Physical Measurements

The elemental analysis of each sample was performed on a Perkin Elmer elemental analyzer (Waltham, MA, USA). IR-spectra were recorded in KBr with a Perkin Elmer Spectrum 2000 FT-IR spectrophotometer, in the 4000–400 cm^−1^ region. Electronic absorption spectra of the ligand and metal complexes were recorded on a Perkin Elmer Lambda-25 spectrometer in DMF within the 200–800 nm range. The molar conductance of the complex in DMF was measured at room temperature using a Crison EC- Meter Basic 30+ conductivity cell (Barcelona, Spain).

### 3.3. Synthesis of the Ligand: (3E)-3-[(2-{(E)-[1-(2,4-Dihydroxyphenyl)ethylidene]amino}ethyl)imino]-1-phenylbutan-1-one (DEPH_2_)

**Scheme 1:**
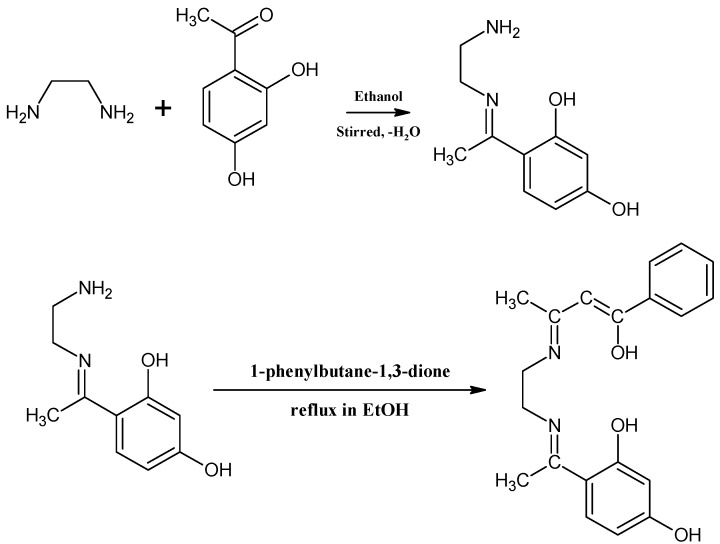
Synthesis of Schiff base ligand (DEPH_2_).

The ligand was prepared by a reported method [17]. A typical procedure for the synthesis of the Schiff base was as follows (Scheme 1): ethylenediamine (0.015 mol, 0.902 g) in ethanol (30 mL) was slowly added to an ethanol solution (40 mL) containing 2′,4′-dihydroxyacetophenone (0.015 mol, 2.282 g), followed by the slow addition of 1-phenylbutane-1,3-dione (0.015 mol, 2.4329 g) dissolved in ethanol (40 mL). The coloured mixture was refluxed with stirring for 4 h, and allowed to stir further at room temperature and the resulting cooled precipitate was filtered, washed severally with ethanol, followed by recrystallization from warm ethanol (Yield = 3.75 g, 74.17%).

### 3.4. General Procedure for the Preparation of the Complexes

The complexes were prepared by the addition of 1.5 mmol of Zn(CH_3_COO)_2_·2H_2_O; Cu(CH_3_COO)_2_·2H_2_O; Ni(CH_3_COO)_2_·4H_2_O; or Co(CH_3_COO)_2_·4H_2_O dissolved in about 20 mL of 40% ethanol solution to a hot ethanolic solution (50 mL) of the ligand (1.5 mmol, 0.5076 g). The colour of the complexes changed in a few minutes. The resulting mixture was then refluxed for 3 h. The precipitated solids were filtered off from the reaction mixture, thoroughly washed with ethanol–water and then with diethyl ether dried over anhydrous calcium chloride.

### 3.5. Antioxidant Assay

#### 3.5.1. 1,1-Diphenyl-2-picrylhydrazyl (DPPH) Radical Scavenging Activity 

DPPH (1,1-diphenyl-2-picrylhydrazyl) radical scavenging activity evaluation is a standard assay in antioxidant activity studies. It is a rapid technique for screening the radical scavenging activity of specific compounds [34]. The free radical scavenging effects of all the complexes and the ligand with DPPH radical were evaluated with various concentrations (100, 200, 300, 400, 500 µg/mL) of the test compounds in 1 mL DMF added to 1.0 mL of 0.4 mM methanol solution of DPPH and vortexed thoroughly. After 30 min incubation period at room temperature in the dark, the scavenging ability that determines the antiradical power of an antioxidant was measured by the decrease in the absorbance of DPPH at 517 nm. Rutin and ascorbic acid (vitamin C) were used as standard drugs. The absorbance decreases when the DPPH is scavenged by an antioxidant, through donation of hydrogen to form a stable DPPH_2_ molecule. All sample tests were performed in three replicates to obtain mean ± S.D. values. The percent of inhibition (I %) of free radical production of DPPH was calculated by using the following equation:

$$\text{DPPH scavenging ability (\%)}=\frac{Abs_{0}-\;Abs_{1}}{Abs_{0}}\;\times \;100$$

where $Abs_{0}$ = the absorbance of the control solution, and $Abs_{1}$ = the absorbance in the presence of sample solutions or standards for positive control.

#### 3.5.2. ABTS: 2,2′-Azinobis-3-ethylbenzothiazoline-6-sulfonic acid Radical Scavenging Assay

The assay of the ABTS scavenging ability of the metal compounds and Schiff base ligand adopted the previously described method [30]. The working solution was prepared by mixing two stock solutions of 7 mM ABTS solution and 2.4 mM potassium persulfate solution in equal amounts (1:1) and allowing the solutions to react in the dark for 12 h at room temperature. The resulting solution was further diluted by mixing ABTS^+^ solution (1 mL) to obtain an absorbance of 0.706 ± 0.001 units at 734 nm using a spectrophotometer. Test samples (1 mL) were allowed to react with the ABTS^+^ solution (1 mL), followed by absorbance reading at 734 nm after 7 min using the spectrophotometer. The ABTS scavenging capacity of the M(II) compounds and Schiff base ligand was compared with that of rutin and butylated hydroxytoluene (BHT) (standards). All test and analysis were run in triplicate and the results obtained were averaged. The percentage inhibition was calculated as ABTS radical scavenging activity using the following equation:

$$\text{(\%) Inhibition}=\frac{Abs_{control}\;-\;Abs_{sample}}{Abs_{control}}\;\times \;100$$

where $Abs_{control}$ is the absorbance of ABTS radical + DMF; $Abs_{sample}$ is the absorbance of ABTS radical + sample (test samples/ standard).

### 3.6. In Vitro Antimicrobial Studies

Antibacterial activity of the samples was determined by the agar well diffusion method [35]. Laboratory strains of six bacterial species which included three Gram-positive bacteria, viz. *Staphylococcus aureus* (ATCC 25923), *Streptococcus faecalis* (ATCC 29212), *Bacillus cereus* (ATCC 10702) and three Gram-negative bacteria *viz*. *Pseudomonas aeruginosa* (ATCC 19582), *Escherichia coli* (ATCC 25922), and *Shigella flexineri* (KZN) were investigated. Ciprofloxacin and amoxicillin were used as the standard antibacterial agents. The bacteria isolates were sub-cultured on nutrient agar plates and incubated at 37 °C for 24 h. A loop full of bacteria cells from the nutrient agar plates was incubated into a nutrient broth (50 mL) in a 250 mL sidearm Erlenmeyer flask and incubated at 37 °C for 18 h with vigorous shaking. Using a sterile glass spreader, 18 h bacterial cultures (100 µL) were used to spread a bacterial lawn on nutrient agar. The Minimum Inhibitory Concentration (MIC) of the ligand and metal complexes were determined using agar dilution method as described by the National Committee for Clinical Laboratory Standards [36]. The bacterial strains were grown at 37 °C overnight and maintained on nutrient agar. Inoculums of the test organisms were prepared in normal saline (9 g L^−1^) compared with 0.5 McFarland standard to attain 5 × 10^5^ (CFU mL^−1^). The suspension was then used to inoculate sterile petri plates of 9.0 cm diameter in which the test organisms were grown. A stock solution of the compounds were prepared in DMSO (Sigma) and further diluted in MHB agar at 50 °C to give a final concentrations ranging from 0.312–10 mg/mL; after pouring into plates and allow the agar to set, plates were inoculated with standardized inocula of the test bacteria, and further incubated at 37 °C for 24 h under aseptic conditions. The MIC was recorded as the lowest concentration at which no visible growth was observed.

## 4. Conclusions

Co(II), Ni(II), Zn(II) and Cu(II) complexes of the unsymmetrical Schiff base ligand derived from 2′,4′-dihydroxyacetophenone and 1-phenylbutane-1,3-dione were synthesized and characterized. Conductance measurements indicate that the complexes are non-electrolytes in solution. The spectral data revealed that the Schiff base acts as a tetradentate ligand, and the Cu(II) and Ni(II) complexes have square planar geometry, while the Co(II) complex has an octahedral geometry. The antimicrobial activity of the ligand and its complexes indicate that the Cu(II) complexes possess higher antibacterial activities than the other complexes and ligand against three Gram-positive bacteria, viz. *Staphylococcus aureus*, *Streptococcus faecalis*, *Bacillus cereus* and three Gram-negative bacteria viz. *Pseudomonas aeruginosa*, *Escherichia coli*, and *Shigella flexineri* in the order Cu(II) > Ni(II) > Co(II) > Zn(II) > DEPH_2_. Such activity is explained on the basis of chelation theory and overtones concept. Further, the antioxidant activity results obtained against free radicals confirmed that the complexes are effective at preventing the formation of the DPPH and ABTS radicals and the lower IC_50_ values observed in antioxidant assays showed that the synthesized complexes exhibited differential and selective effects to scavenge radicals and hence potential as chemotherapeutic drugs to eliminate pathological radical-related diseases from the system.
